# Osteogenesis Imperfecta: New Perspectives From Clinical and Translational Research

**DOI:** 10.1002/jbm4.10174

**Published:** 2019-02-20

**Authors:** Josephine T Tauer, Marie‐Eve Robinson, Frank Rauch

**Affiliations:** ^1^ Shriners Hospital for Children Montreal Quebec Canada

**Keywords:** COLLAGEN TYPE I, FRACTURES, MUSCLE, OSTEOBLAST, OSTEOGENESIS IMPERFECTA, SEQUENCING

## Abstract

Osteogenesis imperfecta (OI) is a monogenic bone fragility disorder that usually is caused by mutations in one of the two genes coding for collagen type I alpha chains, *COL1A1* or *COL1A2*. Mutations in at least 18 other genes can also lead to an OI phenotype. As genetic testing is more widely used, mutations in these genes are also more frequently discovered in individuals who have a propensity for fractures, but who do not have other typical clinical characteristics of OI. Intravenous bisphosphonate therapy is still the most widely used drug treatment approach. Preclinical studies in OI mouse models have shown encouraging effects when the antiresorptive effect of a bisphosphonate was combined with bone anabolic therapy using a sclerostin antibody. Other novel experimental treatment approaches include inhibition of transforming growth factor beta signaling with a neutralizing antibody and the inhibition of myostatin and activin A by a soluble activin receptor 2B. © 2019 The Authors. *JBMR Plus* published by Wiley Periodicals, Inc. on behalf of American Society for Bone and Mineral Research

## Introduction

Osteogenesis imperfecta (OI) is a heritable skeletal disorder that, as the name implies, is caused by defective bone formation.^1^, ^2^ This defect is caused by dominant or recessive mutations that lead to bone fragility and other skeletal manifestations, such as short stature and bone deformities. Extraskeletal tissues and organs can also be involved.^3^ Apart from bone fragility, the classical description of the OI phenotype includes blue or grey discoloration of the sclera and abnormalities of tooth structure called dentinogenesis imperfecta. The prevalence of OI has been estimated at 1 in 13,500 and 1 in 9,700 in two recent population‐based studies from Scandinavia.^4^, ^5^

OI is a rare, but frequently reviewed condition. Several recent general introductions to OI and excellent overviews on the genetic causes and pathomechanisms of OI are available.^1^, ^2^, ^3^, ^6^, ^7^ The present review focuses on recent clinical and translational studies on OI that are of relevance for metabolic bone specialists.

## Clinical Types of Osteogenesis Imperfecta

**Figure 1:**
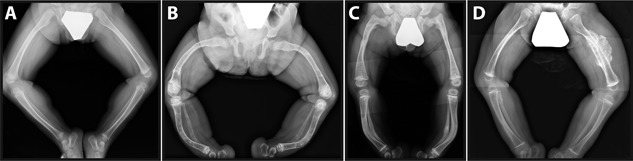
Lower extremity radiographs. (*A*) A 21‐month‐old boy with osteogenesis imperfecta (OI) type I caused by a frameshift mutation in *COL1A1*. (*B*) A 3‐year‐old girl with OI type III caused by a glycine substitution in *COL1A2*. Note the severe bowing of both femurs and tibias, and healing fracture of the distal right femur. (*C*) A 3‐year‐old boy with OI type IV caused by a valine deletion in *COL1A2*. Note the deformities of both femurs and tibias, and healing fracture of the left fibula. (*D*) A 19‐month‐old boy with OI type V caused by a *IFITM5* mutation. Note the hyperplastic callus formation of the left femoral shaft and radiodense metaphyseal bands adjacent to the growth plate.

As the phenotypic severity of OI varies widely, it can be useful to categorize individuals with similar clinical characteristics into more narrowly defined OI types. The 2015 Nosology and Classification of Genetic Skeletal Disorders distinguishes five clinically recognizable OI types (Fig. 1).^8^ OI type I is the mildest phenotype that is usually associated with straight limbs and a body height within or slightly below the reference range; median adult height *Z*‐scores range between −1.1 and −1.5.^9^, ^10^ OI type II represents the most severe end of the phenotypic spectrum and usually leads to death from respiratory failure shortly after birth. OI type III is the most severe form of OI in individuals surviving the neonatal period. Individuals with OI type III almost always have restricted mobility, develop scoliosis, and are very short, with a final height *Z*‐score typically between −8 and −9.^9^, ^10^, ^11^, ^12^, ^13^ The disease severity of OI type IV is intermediate between OI types I and III. With adequate care, most individuals with OI type IV are ambulatory, but more than half develop scoliosis.^12^, ^14^ Short stature is very common in OI type IV, with mean adult height *Z*‐scores between −3.6 and −4.6.^9^, ^10^ The skeletal features of the much rarer OI type V often resemble those of OI type IV, but OI type V is associated with additional distinctive characteristics, such as hyperplastic callus formation (observed in about two‐thirds of patients) and ossification of the interosseous membrane of the forearms (which eventually develops in almost all individuals with OI type V).^15^ Among the various OI types, OI type I is by far the most prevalent, comprising 70% of the entire cohort in a population‐based study.^4^

In addition to OI types I to V, many higher‐number OI types have been proposed based on genetic test results rather than phenotypic features. For example, the Online Mendelian Inheritance of Man database lists a new OI type for each newly discovered gene that is linked to an OI phenotype (http://www.ncbi.nlm.nih.gov/omim/). The drawback of this approach is that recoding the involved gene as an OI type with an arbitrary number adds a layer of complexity to the classification without providing additional information; it would be simpler to just state the name of the gene involved. Describing OI by a combination of the clinical OI phenotype (I to V) and the affected gene as proposed by the 2015 Nosology and Classification of Genetic Skeletal Disorders,^(8)^ provides more useful information to clinicians.

## Genetic Causes of Osteogenesis Imperfecta

The majority of individuals with OI have a disease‐causing mutation in one of the two genes that code for collagen type I alpha chains, *COL1A1* and *COL1A2*. Collagen type I is the main component of the organic bone matrix and therefore plays a key role in the integrity of bone tissue.^1^, ^2^, ^6^ Mutations in 18 genes other than *COL1A1* and *COL1A2* have been associated with OI phenotypes and are listed in the OI mutation database (https://oi.gene.le.ac.uk). These genes are all expressed in osteoblasts, and most of them are directly involved in collagen type I metabolism, even though some of these genes seem to play a role in other aspects of osteoblast function such as Wnt signaling.^1^ Defects in the newer OI‐related genes usually lead to recessive forms of OI, but two genes (*IFITM5*, *P4HB*) are associated with dominant OI, and two genes (*PLS3*, *MBTPS2*) lead to X‐linked bone fragility.

Almost all individuals with a typical clinical presentation of OI have a detectable mutation in one of the currently known OI‐associated genes. A recent sequencing study in close to 600 individuals with a clinical diagnosis of OI found disease‐causing mutation in 97% of patients with OI type I (all of whom had *COL1A1* or *COL1A2* mutations) and in 99% of individuals with the more severe OI types (77% had *COL1A1* or *COL1A2* mutations).^16^ Thus, even though some OI genes remain to be discovered, they can be expected to affect only a small number of individuals with a typical OI phenotype.

Regarding genotype–phenotype correlations, *COL1A1* mutations leading to haploinsufficiency of the collagen type I alpha 1 chain consistently give rise to OI type I, with mild bone fragility, blue/grey sclera, and normal‐looking teeth. Haploinsufficiency can result not only from *COL1A1* stop or frameshift mutations,^17^ but also from some splice site mutations and deletions of the entire *COL1A1* gene.^18^, ^19^ These mutations lead to decreased collagen type I production by osteoblasts and other cells and therefore have also been called quantitative collagen mutations.^17^ Mutations that change the amino acid sequence of the collagen type I alpha chains can be called qualitative mutations. They are frequently caused by glycine substitutions in the triple helical domain of the alpha 1 or alpha 2 chains. Such glycine substitutions can cause the entire range of phenotypic severity of OI, from mild to lethal.^20^

Mutations in genes other than *COL1A1* and *COL1A2* are usually associated with a moderate to very severe phenotype (OI type II, III, IV, or V). However, there are some exceptions. Some recessive *BMP1* mutations are associated with a mild disease course that is similar to OI type I.^21^ *PLS3* mutations also lead to a clinical picture that can resemble OI type I.^22^

## Genetic Testing for Osteogenesis Imperfecta

Elucidating the disease‐causing mutation is useful in patients who have a clinical diagnosis of OI, as it provides information about the risk of recurrence in a family and allows for the identification of affected family members. Genetic testing can also have implications for clinical management. For example, finding the OI type V specific *IFITM5* mutation indicates that the patient has a high risk of developing hyperplastic callus,^23^ radial head dislocation,^24^ and abnormalities in the cranio–cervical junction.^25^ Mutations affecting the C‐propeptide of the collagen type I alpha 1 chain are frequently associated with hip dysplasia,^26^ and glycine substitutions caused by mutations in exon 49 of *COL1A2* may predispose to intracranial hemorrhage.^27^

Genetic testing can also be useful when the diagnosis is not obvious from the clinical picture. For example, it can sometimes be difficult to distinguish OI type I from other causes of recurrent fractures in children and adolescents.^28^ This situation was investigated in a study of 94 individuals less than 21 years of age who had a significant fracture history (one or more long‐bone fracture of the lower extremities, two or more long‐bone fractures of the upper extremities, one or more vertebral compression fracture: all in the absence of major trauma), but had white sclera and no signs of dentinogenesis imperfecta; therefore, they did not have unequivocal signs of OI.^29^ Sequence analysis of a panel of OI‐associated genes found disease‐causing mutations in 26 (28%) of these individuals. Hence, a proportion of children and adolescents with recurrent fractures have OI even if the family history is negative and the phenotypic appearance does not clearly suggest a diagnosis of OI.

As genetic testing is more widely used in research and clinical practice, it is becoming apparent that individuals with a typical OI phenotype only represent the severe end of the spectrum of bone disorders that are caused by mutations affecting collagen type I. As noted, “typical OI mutations” (glycine substitutions in the triple helical domain of the collagen type I alpha 1 and alpha 2 chains) are far more frequent in exome sequencing databases than is expected from the population prevalence of OI, suggesting that the majority of OI mutations do not lead to a readily recognizable OI phenotype.^30^ Examples for this are two *COL1A2* mutations (p.Gly496Ala and p.Gly703Ser) that are found in the Icelandic population with relatively high frequency.^31^ These mutations are associated with low BMD, fractures, and a slightly below‐average height, but not with other clinical features of OI, such as dentinogenesis imperfecta or blue/grey sclera. Another study has shown that some glycine substitutions caused by *COL1A2* mutations can have such mild effects that they do not cause a detectable phenotype in the heterozygous state, but lead to OI only when homozygous.^32^ It is likely that more examples of partial OI phenotypes will be found when sequencing studies are more frequently performed in adults with a diagnosis of osteoporosis.

Because OI usually is associated with low bone mass, one might choose to limit sequencing for OI mutations to individuals with low BMD. However, this strategy would fail to correctly identify some individuals with OI. Mutations that affect the C‐propeptide cleavage site are associated with frequent fractures in the presence of elevated BMD.^33^ In a series of 29 individuals with C‐propeptide cleavage site mutations, the mean lumbar spine BMD *Z*‐score was +2.9, whereas a control group with *COL1A1* haploinsufficiency mutations had a mean lumbar spine BMD *Z*‐score of −2.2.^34^ Similar observations have been made in patients with mutations affecting BMP1, the enzyme that is responsible for C‐propeptide cleavage.^21^ These findings highlight the utility of sequence analysis of OI genes in young individuals with recurrent low‐trauma fractures, even if BMD is normal or elevated.

## Fractures

The high fracture incidence in OI has been recognized since the first medical description of the disorder in 1690,^35^ but the fracture epidemiology across the lifespan has been described only recently. A population‐based study in Denmark found that individuals with a diagnosis of OI have eightfold higher fracture rates (all skeletal sites combined) than the general population.^36^ The relative fracture risk in OI as compared to the general population varied considerably with age (11‐fold higher in individuals with OI below 20 years of age, 6‐fold from 20 to 54 years, and 4‐fold in individuals aged 55 years and higher). As in the general population, women with OI had a higher fracture incidence during menopause than premenopausal women. OI was associated with a particularly high relative risk of femur and lower leg fractures, suggesting that fractures are not only more frequent in OI, but also more severe than in the general population. This study did not have information on OI types, but given that a population‐based cohort was investigated, the majority of study participants must have had OI type I. A limitation of the study is that spine radiographs were not obtained systematically; it is therefore likely that the incidence of vertebral fractures was underestimated.

### Atypical femur fractures?

A study in children and adolescents with OI type I caused by *COL1A1* haploinsufficiency mutations suggested that the rate of femur and tibia fractures was about 90 times higher than in their healthy peers.^37^ In adults with OI, it has been estimated that the incidence of femoral shaft fractures is about 35 times higher than in the general population.^38^

Diaphyseal femur fractures are thus common in OI. Such fractures often occur after minimal trauma, are often transverse, are almost always complete, and are non‐ or minimally comminuted, thereby fulfilling four major criteria for establishing a diagnosis of atypical femur fractures, as defined for fractures that occur in the context of postmenopausal osteoporosis.^39^ By extension, some case reports and small case series on OI have recently labeled transverse femur fractures as “atypical femur fractures,” especially when they occurred after bisphosphonate use.^40^

**Figure 2:**
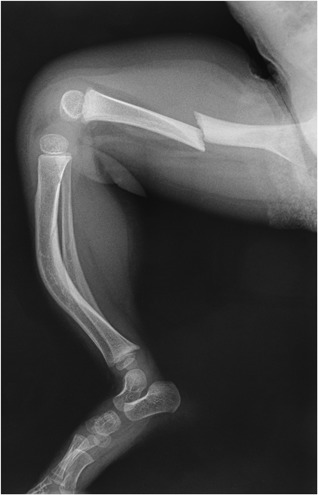
Lateral view of a diaphyseal femur fracture in a 21‐month‐old boy with osteogenesis imperfecta type IV, without previous deformities of the femur and in the absence of prior bisphosphonates treatment.

However, transverse diaphyseal femur fractures have been one of the most common types of fracture in OI even before the bisphosphonate era (Fig. 2).^41^ We found that about 25% of fractures occurring in the nondeformed femurs of individuals with OI fulfill the criteria of atypical femur fractures, regardless of whether they had been exposed to bisphosphonates.^42^ Overall, systematic studies on more than 300 femur fractures have failed to detect a relationship between bisphosphonate use and diaphyseal femur fractures in OI.^42^, ^43^, ^44^ These studies concurred that the main risk factor for such femur fractures in OI was the severity of the underlying disease rather than bisphosphonate treatment history. We submit that labeling diaphyseal femur fractures that are common in OI as “atypical” because they are rare in the context of postmenopausal osteoporosis is confusing and should be avoided.

## Other Disease Manifestations

Collagen type I is found in many tissues; therefore, collagen type I mutations might affect those tissues directly. In addition, abnormal bone shape and restricted mobility can lead to secondary problems in extraskeletal tissues. Thus, it is not surprising that many organ systems can be directly or indirectly affected in individuals with OI.^3^ Here we focus on a few topics that have been highlighted by recent studies.

A population‐based study in Denmark showed that individuals with OI have an increased risk of death at any age, leading to decreased life expectancy.^45^ The median age of death was 72.4 years for males and 77.4 years for females with OI, which was 10 and 7 years, respectively, earlier than death in the control population. In particular, the OI group had a higher risk of death from respiratory disease, gastrointestinal (GI) disease, and trauma. Although death after trauma can be assumed to be related to fractures, respiratory and GI disease may not be directly linked to bone fragility. The exact nature of these respiratory and GI disorders could not be determined in that study, but the authors speculated that increased use of nonsteroidal anti‐inflammatory drugs could play a role in GI disorders. Another common GI problem in OI is chronic constipation that can be severe in patients with pelvic deformity because of acetabular protrusion.^46^, ^47^, ^48^ Respiratory issues are a well‐known problem in the context of OI. Pulmonary hypoplasia and parenchymal abnormalities are thought to be the cause of death in OI type II.^49^, ^50^ Lung histology is also abnormal in several mouse models of OI.^51^, ^52^, ^53^ In addition, lung function can be affected by scoliosis.^52^, ^54^, ^55^

Sleep apnea, a disorder characterized by pauses in breathing, affects 1% to 6% of adults and 2% of children in the general population.^56^ Sleep apnea can lead to serious health issues, such as cardiovascular disease; through increased daytime sleepiness, it increases the risk of accidents. Sleep apnea appears to be more frequent in OI. A web‐based survey found that self‐reported sleep apnea was present in 32% of adults with severe OI, in 17% with moderate OI, and in 9% with mild OI.^57^ In a Finnish study, 15% of adults with OI (all types combined) had “diagnosed sleep apnea and used a positive airway pressure ventilator during sleep.”^58^ In a French cohort of 188 children with OI, 6.4% had sleep‐disordered breathing as documented by polysomnography.^59^ Thus, sleep apnea may be a potentially serious, but little‐studied aspect of OI.

Dental and craniofacial issues are also a source of major concern in OI. In a recent survey that largely included more severely affected individuals with OI, 75% of respondents noted that dental and craniofacial issues impacted their quality of life.^57^ A frequent dental abnormality in OI is dentinogenesis imperfecta, which is caused by dysplastic dentin and can lead to dental discoloration, tooth fracture, and attrition.^60^ Genotype–phenotype correlation studies show that the large majority of patients with OI types III and IV caused by qualitative mutations have dentinogenesis imperfecta, whereas only a small minority of individuals with *COL1A1* haploinsufficiency mutations have dentinogenesis imperfecta that is visible on clinical inspection.^37^, ^61^, ^62^, ^63^ Beyond dentinogenesis imperfecta, tooth agenesis is a common finding in OI.^64^ These dental abnormalities may contribute to dysplasia of the mandible and maxilla, which frequently lead to malocclusion in individuals with OI types III or IV.^65^, ^66^

Cranial base abnormalities are an important complication of OI and can lead to compression of the structures of the posterior fossa, Chiari malformation, spinal cord syrinx formation, and hydrocephalus.^67^, ^68^ Such abnormalities are uncommon in mild OI, but develop in more than half of individuals with OI type III.^25^, ^69^ Bisphosphonate treatment may slow down the development of cranial base abnormalities, but it does not seem to prevent this complication.^25^, ^70^

Several studies have shown that the muscle system is involved in OI in humans and in mouse models.^71^ Muscle mass is decreased in children and adolescents with OI, even when their smaller body size is taken into account.^72^ Dynamic muscle tests in children with OI type I and IV have revealed functional deficits that cannot be explained by low muscle mass alone.^73^, ^74^ There are many potential reasons for muscle deficits in OI, including a direct effect of collagen type I mutations on muscle,^75^ joint hyperlaxity,^76^ and physical inactivity.

## Treatment

The clinical management of OI depends on the severity of the phenotype. In uncomplicated OI type I, physical activity may be similar to the general population.^77^ Treatment needs may be limited to fracture management with the main purpose of medical follow‐up to screen for complications such as vertebral compression fractures, which, if present, may be an indication for i.v. bisphosphonate treatment.^30^, ^78^, ^79^ In more‐severe OI, where long‐bone deformities, scoliosis, and reduced mobility are major concerns, a multidisciplinary orthopedic and rehabilitation intervention program may be required.^80^

Ensuring adequate vitamin D intake is often recommended,^81^, ^82^ but how much vitamin D intake is needed in OI is not well‐established. A recent randomized controlled trial compared two doses of vitamin D supplementation, 400 IU and 2000 IU, in 60 children with OI, most of whom were vitamin D sufficient at baseline [mean serum 25(OH) vitamin D concentration: 67 nmol/L].^83^ No differences in lumbar spine areal BMD or any other outcome measures other than 25(OH) vitamin D serum levels were found between the two doses of vitamin D supplementation.

### Bisphosphonates

Bisphosphonate therapy is the most widely used medical treatment in OI. The use of bisphosphonates in OI has been the focus of several recent reviews^30^, ^84^, ^85^; therefore, only a brief summary is given here. All studies agree that bisphosphonate treatment increases BMD in individuals with OI. However, the few randomized controlled trials that have been performed on bisphosphonate treatment in OI were not powered to assess outcomes other than BMD and had short treatment durations. Systematic reviews of randomized trials in OI consequently are unanimous in their verdict that there is not enough evidence to judge whether bisphosphonate therapy improves outcomes other than BMD.^86^, ^87^, ^88^ Nevertheless, long‐term follow‐up data from observational studies provide some information on clinically relevant treatment outcomes.

**Figure 3:**
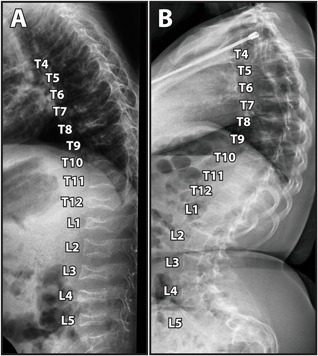
Lateral spine radiograph of a girl with osteogenesis imperfecta type IV showing reshaping of vertebral bodies during bisphosphonate treatment. (*A*) Age 2 years. (*B*) Age 15 years.

When i.v. bisphosphonate treatment is given to growing children, vertebra with deformities from compression fractures can gain a more normal shape through growth (Fig. 3).^79^, ^89^, ^90^, ^91^ The potential for vertebral reshaping depends on how much growth remains at the time when bisphosphonate treatment is started. In a study that assessed patients who had their first bisphosphonate infusion before 5 years of age, the majority of compressed vertebra had regained a normal shape by the time the children had reached final height.^91^ However, despite the positive effect on the shape of individual vertebra, bisphosphonates do not seem to have a major effect on the development of scoliosis. Two large studies found that i.v. bisphosphonate treatment slowed down the progression rate of scoliosis in the most severely affected patients, but the prevalence of scoliosis at maturity was not influenced by bisphosphonate treatment history.^12^, ^14^

Bisphosphonate treatment seems to decrease long‐bone fracture rates by 30% to 60% in children with OI.^78^, ^91^, ^92^, ^93^ Given the high baseline fracture rates in OI, this means that many long‐bone fractures occur despite bisphosphonate treatment. Nevertheless, it has been observed that i.v. bisphosphonate treatment can improve mobility, especially when started early in life.^79^, ^90^ Long‐term follow‐up suggests that most children with OI type IV, but not those with OI type III, achieve the ability to walk independently.^11^

Regarding potential adverse events of bisphosphonates, delayed healing of osteotomy sites is frequently observed in children with OI who have intramedullary rodding procedures. Intravenous bisphosphonate therapy appears to increase the risk for this complication.^94^ Avoiding bisphosphonate treatment in the 4 months following surgery seems to decrease the percentage of osteotomy sites that heal with delay or not at all.^95^ Another potential adverse event linked to bisphosphonate therapy is osteonecrosis of the jaw. However, systematic reviews did not identify any confirmed occurrence of this problem in OI.^96^, ^97^

### Drugs other than bisphosphonates

Many individuals with OI have fractures despite bisphosphonate treatment. Long‐bone deformities, short stature, and scoliosis continue to occur in children with severe OI even if treatment is started in infancy.^12^, ^91^ More effective treatment options are therefore needed.

Denosumab is an antiresorptive drug that uses an antibody against RANKL to inhibit osteoclast differentiation and activity.^98^ Denosumab is approved for the treatment of postmenopausal osteoporosis and other skeletal disorders in adults. Studies in children with OI are being conducted. A few published case series on denosumab treatment in children with OI found a decrease in bone metabolism markers and an increase in BMD.^99^, ^100^

Compared to bisphosphonates, denosumab has a much shorter duration of action, but it is not clear how long the antiresorptive effect of denosumab persists in children. The clearance of denosumab seems to depend on the amount of available RANKL.^101^ The amount of RANKL produced by children is not well‐established. Once the antiresorptive effect of a denosumab injection has run its course, hypercalcemia can develop very quickly in children, as evidenced in an 8‐year‐old girl who received denosumab for juvenile Paget's disease and who had a very high serum calcium concentration only days after a blood test had shown normocalcemia.^102^ Hypercalcemia and hypercalciuria have also been reported in children with OI receiving denosumab.^103^ It is possible that the long‐term antiresorptive action of a bisphosphonate is still needed in children receiving denosumab to prevent intermittent hypercalciuria and hypercalcemia and to prevent rapid bone loss once denosumab is discontinued.

Teriparatide, a bone anabolic agent, has been assessed in a randomized controlled trial in adults with OI.^104^ This showed increased BMD in mild OI, but was less effective in moderate‐to‐severe OI. In accordance with these findings, positive effects of teriparatide were observed in two studies that focused on adults with OI type I.^105^, ^106^ It therefore appears that teriparatide can be useful for the treatment of mild OI in adults. However, the use of teriparatide in children is contraindicated, as studies in growing rats have shown an increased risk of osteogenic sarcoma.^107^

### Drugs on the horizon

A variety of novel pharmaceutical compounds have been assessed, mainly in preclinical studies. The OI mouse models that have been used for most of these studies are summarized in Table 1.

**Table 1:** Mouse Models of Osteogenesis Imperfecta Used for Preclinical Studies of Novel Drug Treatments

Mouse	Gene	Nucleotide	Protein	Bone phenotype
Dominant				
*Brtl* 127	*Col1a1*	c.1546G>T	Triple‐helical glycine substitution (p.Gly349Cys)	30% perinatal lethality, small body size, rib fractures, long‐bone deformity, bone fragility, reduced BMD; increased bone turnover because of increased osteoclast precursors and reduced osteoblast activity
*Jrt* 128	*Col1a1*	Splice mutation	Exon 9 skipping (deletion of 18 triple‐helical amino acids)	Small body size, short long‐bones, low BV/TV and BMD, spontaneous fractures; reduced tensile properties in the skin, tail tendon tissue reduced
*G610C* 129	*Col1a2*	c.2098G>T	Triple‐helical glycine substitution (p.Gly610Cys)	Moderately severe phenotype, depending on genetic background. Reduced body size, BV/TV, BMD and bone strength
*oim*+/−130	*Col1a2*	c.3983delG	proα2(I) decreased by 50%	Intermediate between *oim‐/‐* and wild‐type; normal body size, normal cortical thickness, lower mechanical strength
				
Recessive				
*oim*−/−131, 132	*Col1a2*	c.3983delG	proα2(I) absent	Small body size, fractures, limb deformities, cortical thinning, joint laxity, osteopenia, reduced BV/TV and BMD, kyphosis, increased osteoclast activity
*Crtap−/−* 133, 134	*Crtap*		CRTAP absent	Moderate phenotype: growth delay, skeletal deformity, kyphosis, reduced BV/TV but high bone mineralization, cartilage dysplasia, decreased material properties of the skin

+/− = heterozygous for the mutation; −/− = homozygous for the mutation; BMD = bone mineral density; BV/TV = bone volume per tissue volume.

Antibody‐mediated sclerostin inhibition is an approach to stimulate bone formation that has been used in clinical studies to treat osteoporosis in adults.^98^ Regarding OI, sclerostin antibody treatment increased bone mass and strength in several mouse models such as *Brtl*, *G610C*, and *Crtap‐/‐*,^108^, ^109^, ^110^ but was less beneficial in the *Jrt* mouse.^111^ Short‐term treatment with sclerostin antibody stimulated bone formation, suppressed bone resorption, and increased BMD in a small group of adults with OI.^112^

One issue with short‐acting treatment agents such as sclerostin antibody is that the bone will rapidly revert towards its baseline status once the treatment is discontinued, as has been noted in adults with osteoporosis.^113^ In the *Brtl* mouse, bone loss after the discontinuation of sclerostin antibody treatment could be prevented by subsequent administration of a bisphosphonate.^114^ It might also be advantageous to give bisphosphonates concomitant with sclerostin antibody, at least during growth. Studies in both *G610C* and *Brtl* mice have shown that the metaphyseal trabecula of growing mice were retained through the antiresorptive action of bisphosphonates and could serve as templates for the formation of new bone that was stimulated by sclerostin inhibition.^115^, ^116^

Transforming growth factor beta (TGFβ) plays an important role in determining bone mass and quality.^117^ Mice overexpressing TGFβ in bone have osteoporosis,^118^ whereas mice with genetic TGFβ inhibition have stronger bones.^119^ TGFβ signaling is increased in *Crtap‐/‐* and *G610C* mice, and pharmacologic inhibition of TGFβ with a neutralizing antibody led to higher bone mass and stronger bones.^120^ TGFβ inhibition also improved the histological abnormalities of lung tissue in *Crtap‐/‐* mice. *Jrt* mice also seem to have increased TGFβ signaling in bone tissue, but treatment with TGFβ neutralizing antibody did not have an obvious effect on bones or lungs.^53^, ^121^ It therefore appears possible that the effect of TGFβ inhibition in OI varies with the disease‐causing mutation and the related bone phenotype.

The muscle phenotype that is seen in human OI is replicated in some mouse models, such as *oim* and *Jrt* mice.^71^ Given the close correlation between muscle and bone,^122^ this led to the hypothesis that treatments targeting muscle could be beneficial for both muscle and bone in OI. Myostatin and activin A are two members of the TGFβ family that inhibit muscle growth by signaling through the activin receptor 2B.^123^ A soluble activin receptor 2B that inhibits both myostatin and activin A led to a marked increase in muscle and bone mass in *oim* mice.^124^ A slightly different soluble activin receptor 2B had similarly beneficial effects on muscles and bones in *oim* and *G610C* mice.^125^, ^126^ Inhibition of myostatin and activin A warrants further exploration as novel treatment approaches in OI.

## Disclosures

The authors state that they have no conflicts of interest.
